# Supplementary material for: Synaptic connectivity to L2/3 of primary visual cortex measured by two-photon optogenetic stimulation
Source: eLife. 2022 Jan 21;11:e71103. doi: 10.7554/eLife.71103 (PMC8824465; doi:10.7554/eLife.71103)
Supplement: Transparent reporting form [file elife-71103-transrepform1.docx]

***eLife’s* transparent reporting form**

We encourage authors to provide detailed information *within their submission* to facilitate the interpretation and replication of experiments. Authors can upload supporting documentation to indicate the use of appropriate reporting guidelines for health-related research (see [EQUATOR Network](http://www.equator-network.org/%20)), life science research (see the [BioSharing Information Resource](https://biosharing.org/)), or the [ARRIVE guidelines](http://www.plosbiology.org/article/info:doi/10.1371/journal.pbio.1000412) for reporting work involving animal research. Where applicable, authors should refer to any relevant reporting standards documents in this form.

If you have any questions, please consult our Journal Policies and/or contact us: [editorial@elifesciences.org](mailto:editorial@elifesciences.org).

**Sample-size estimation**

- You should state whether an appropriate sample size was computed when the study was being designed
- You should state the statistical method of sample size computation and any required assumptions
- If no explicit power analysis was used, you should describe how you decided what sample (replicate) size (number) to use

Please outline where this information can be found within the submission (e.g., sections or figure legends), or explain why this information doesn’t apply to your submission:

We did not estimate sample size during study design. We probed >900 connections onto L2/3 pyramidal cells from each of the following Cre lines: Penk, Rorb, Tlx3, Ntsr1, Pvalb, and Sst. Fewer connections were probed using Scnn1a Cre line due to the relatively sparse opsin expression in this line. Similarly, relatively few connections were probed to subclasses of interneurons due to their relatively low density. These cases are acknowledged in the Results. The number of connections probed within given pre- and postsynaptic cell classes is comparable to or (in most cases) much greater than the number of connections sampled in previous studies.

**Replicates**

- You should report how often each experiment was performed
- You should include a definition of biological versus technical replication
- The data obtained should be provided and sufficient information should be provided to indicate the number of independent biological and/or technical replicates
- If you encountered any outliers, you should describe how these were handled
- Criteria for exclusion/inclusion of data should be clearly stated
- High-throughput sequence data should be uploaded before submission, with a private link for reviewers provided (these are available from both GEO and ArrayExpress)

Please outline where this information can be found within the submission (e.g., sections or figure legends), or explain why this information doesn’t apply to your submission:

The number of connections probed and the number of recorded (i.e. postsynaptic) neurons are stated in the figure legends. Quality control criteria for analysis of individual sweeps are described in Methods, *Analysis of PSP amplitudes* section.

**Statistical reporting**

- Statistical analysis methods should be described and justified
- Raw data should be presented in figures whenever informative to do so (typically when N per group is less than 10)
- For each experiment, you should identify the statistical tests used, exact values of N, definitions of center, methods of multiple test correction, and dispersion and precision measures (e.g., mean, median, SD, SEM, confidence intervals; and, for the major substantive results, a measure of effect size (e.g., Pearson's r, Cohen's d)
- Report exact p-values wherever possible alongside the summary statistics and 95% confidence intervals. These should be reported for all key questions and not only when the p-value is less than 0.05.

Please outline where this information can be found within the submission (e.g., sections or figure legends), or explain why this information doesn’t apply to your submission:

The statistical tests utilized are reported alongside the exact p-value and sample sizes throughout the Results section. Individual data points are presented in all primary figures except Figure 1. Corresponding data points are shown in Figure 1-figure supplement 1, 2, and 3. Violin plots are used to summarize the distributions of PSP amplitudes and rise times in Figure 7A,C. Individual data points for most categories of connection are plotted with regard to spatial location elsewhere in the Figure (7D,E). Underlying data points for all categories of connection are included in the supporting data file.

All representations of connection rate, divergence probability, and convergence probability include 95% confidence intervals. Representations of mean values include SD, representations of median values include interquartile ranges.

(For large datasets, or papers with a very large number of statistical tests, you may upload a single table file with tests, Ns, etc., with reference to sections in the manuscript.)

**Group allocation**

- Indicate how samples were allocated into experimental groups (in the case of clinical studies, please specify allocation to treatment method); if randomization was used, please also state if restricted randomization was applied
- Indicate if masking was used during group allocation, data collection and/or data analysis

Please outline where this information can be found within the submission (e.g., sections or figure legends), or explain why this information doesn’t apply to your submission:

Groups were determined by transgenic Cre line, method of opsin expression (transgenic or viral), the locations of pre- and postsynaptic cells relative to: i) the pia and ii) each other. Classification of postsynaptic cells by intrinsic physiological properties is described in the Methods, *Electrophysiological recordings* section and Figure 3-figure supplement 1. The nature of the grouping precluded masking during data collection and analysis. However, quantitative measures of connectivity (Figure 5 –figure supplement 2, Figure 6 – figure supplement 1) and connection properties (Figures 7-8) were agnostic to grouping.

**Additional data files (“source data”)**

- We encourage you to upload relevant additional data files, such as numerical data that are represented as a graph in a figure, or as a summary table
- Where provided, these should be in the most useful format, and they can be uploaded as “Source data” files linked to a main figure or table
- Include model definition files including the full list of parameters used
- Include code used for data analysis (e.g., R, MatLab)
- Avoid stating that data files are “available upon request”

Please indicate the figures or tables for which source data files have been provided:

Source data corresponding to graphs in Figures 1-10 are included as source data files. Jupyter notebooks to generate the primary figures have been shared at <https://github.com/travis-open/twop_opto_data>. Neurodata without borders (nwb) files containing original electrophysiological recordings are archived as a Dryad Digital Repository. <https://datadryad.org/stash/share/1aykN8S3HOGEPhFIkzVFq3pmCVNawzFRTMs5BJxCY9s%2FOPZxpv1iyJM%3D&reserved=0>
